# Supplementary material for: Basal condensation of Numb and Pon complex via phase transition during Drosophila neuroblast asymmetric division
Source: Nat Commun. 2018 Feb 21;9:737. doi: 10.1038/s41467-018-03077-3 (PMC5821850; doi:10.1038/s41467-018-03077-3)
Supplement: Supplementary file 1 — Supplementary Information [file 41467_2018_3077_MOESM1_ESM.pdf]

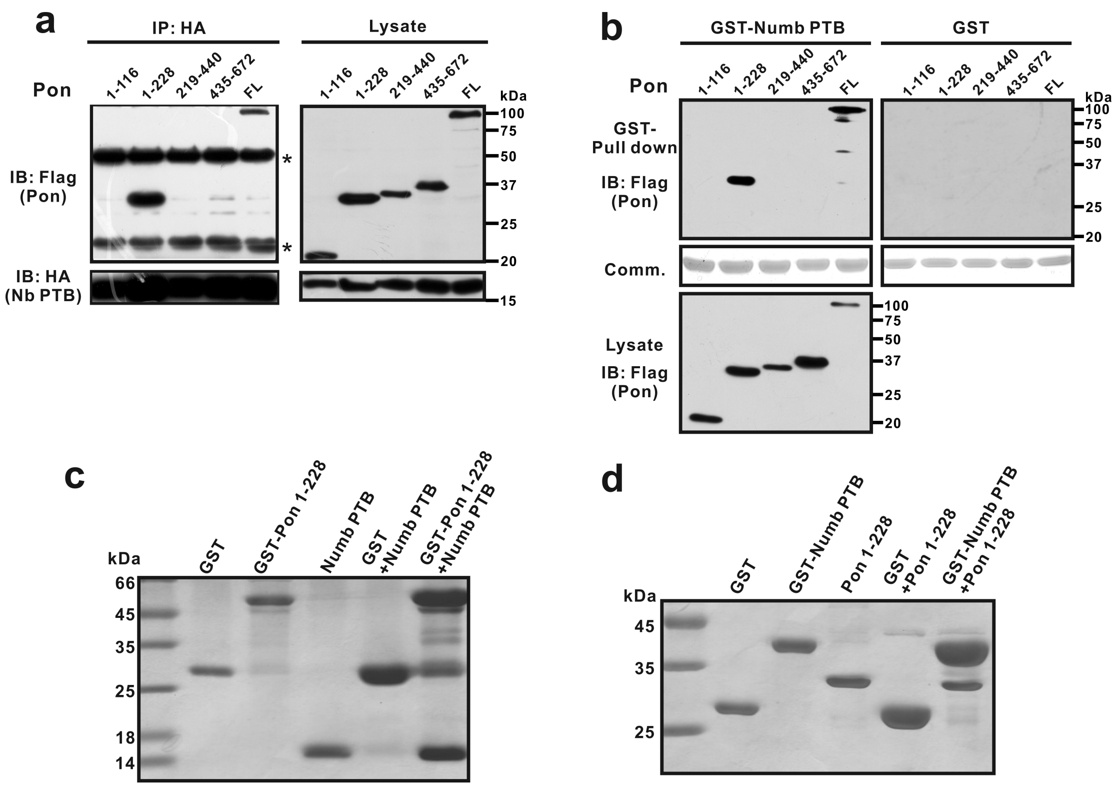

Supplementary Figure 1 *Drosophila* Numb PTB interacts with Pon N-terminal fragment. (**a**) HEK293T cells were co-transfected with HA-Numb PTB and Flag-Pon full length (FL) protein or various fragments. Only Flag-Pon FL or Flag-Pon1-228 could be Co-IPed by HA-Numb PTB. (**b**) HEK293T cell lysate GST pull-down assay showed that GST-Numb PTB but not the GST control interacts with Flag-Pon FL and Flag-Pon1-228. In contrast, Flag-Pon1-116 could not be pulled down by GST-Numb PTB. (**c-d**) GST pull-down assay showed that more than 1:1 molar ratio of Numb PTB could be pulled down by GST-Pon1-228 (**c**), whereas less than 1:1 molar ratio of Pon1-228 could be pulled down by GST-Numb PTB (**d**). In this assay, GST or GST-fusion proteins were incubated with 1:3 molar ratios of target proteins. Uncropped blots are shown in Supplementary Fig. 10.

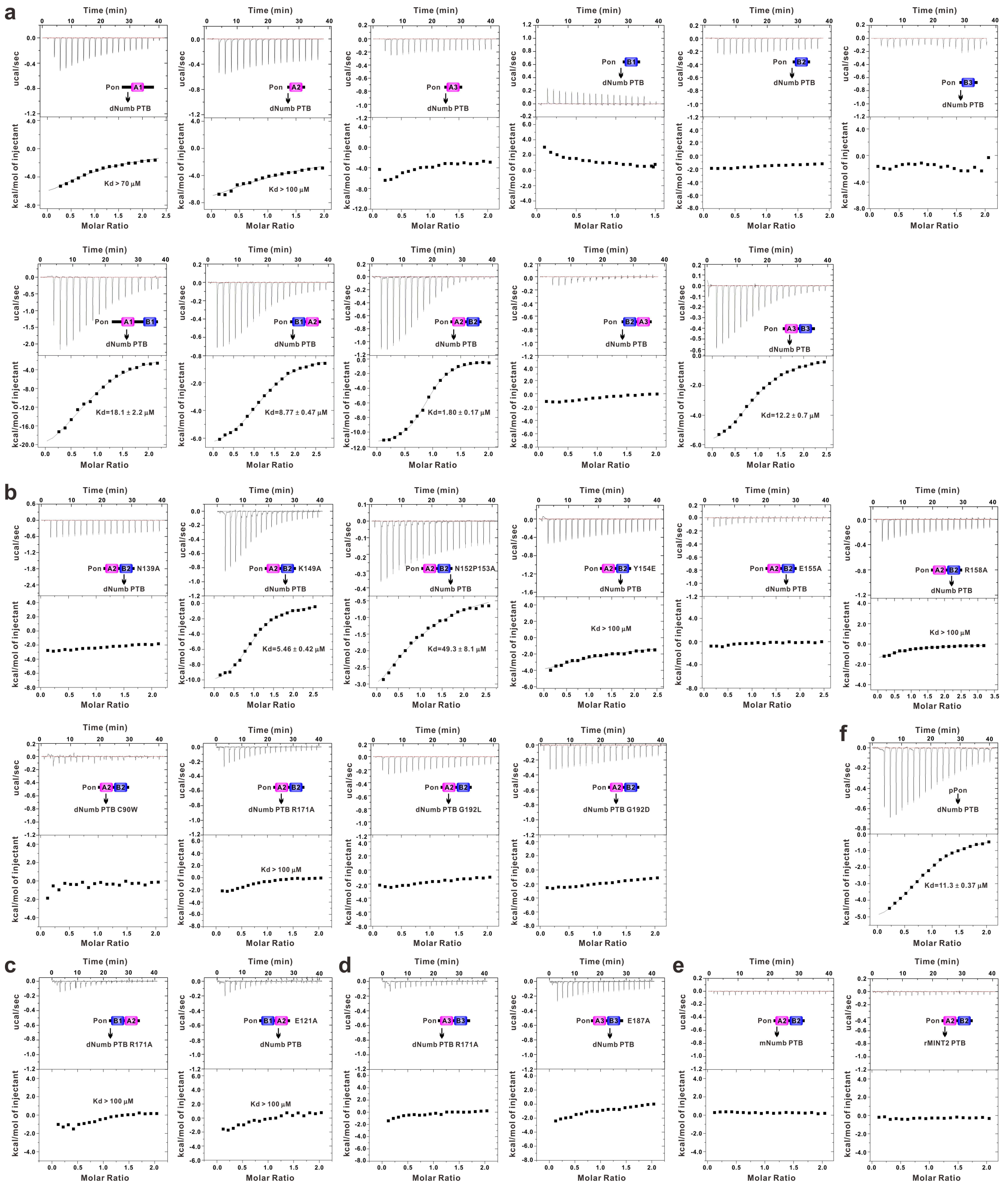

Supplementary Figure 2 ITC-based mapping and validation of the interaction between *Drosophila* Numb PTB and Pon fragments. (**a**) ITC-based measurement of the binding affinities of *Drosophila* Numb PTB and Pon fragments. (**b**) The site-directed mutations on *Drosophila* Numb PTB or Pon A2B2 based on the crystal structure disrupted or impaired their interaction. (**c-d**) Validation of the interaction between *Drosophila* Numb PTB and Pon B1A2 (**c**) or Pon A3B3 (**d**). (**e**) Pon A2B2 can not bind to mouse Numb PTB or rat MINT2 PTB. (**f**) pPon binds to *Drosophila* Numb PTB.

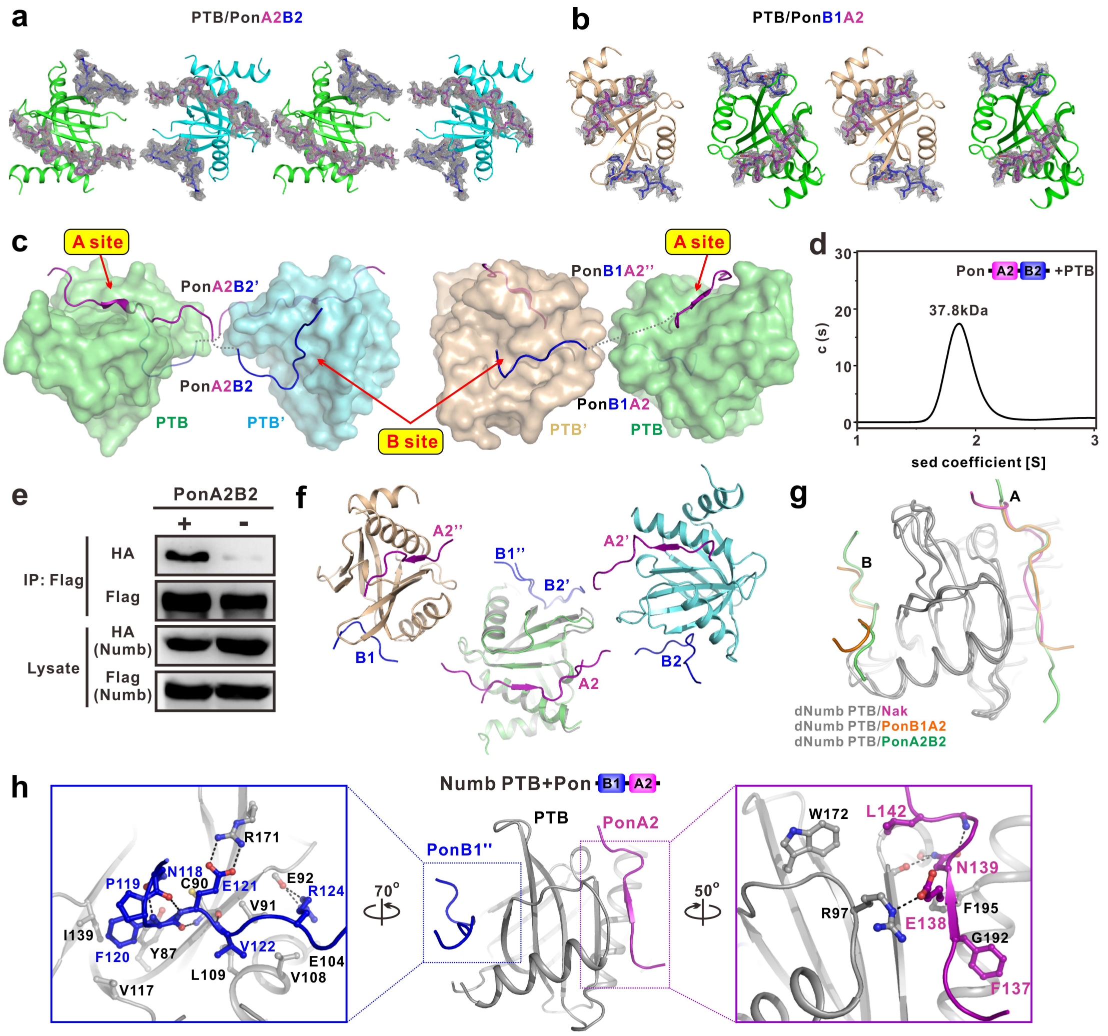

Supplementary Figure 3 The multivalent interaction between *Drosophila* Numb PTB and Pon. (**a&b**) Stereo images of the omit maps for PTB/Pon A2B2 (**a**) and PTB/Pon B1A2 (**b**) complexes contoured at 1.0 σ. (**c**) Ribbon and surface representations of Numb PTB/Pon A2B2 (left) and Numb PTB/Pon B1A2 (right) complexes as viewed from the side. The A and B sites are indicated. (**d**) Pon A2B2 binding-induced dimerization of Numb PTB in solution. SV experiments of Numb PTB in the presence of three molar ratios of Pon A2B2. The SV profile of PTB/A2B2 displays as a major peak corresponding to a dimer-of-dimers (37.8 kDa). The theoretical molecular weights of PTB and A2B2 are 15.7 and 3.7, respectively. (**e**) Pon A2B2 binding-induced dimerization of full-length Numb. HEK293T cells were transfected with HA-Numb and Flag-Numb. HA-Numb could only be Co-IP'ed by Flag-Numb in the presence of Pon A2B2. (**f**) Superimposition of Numb PTB/Pon A2B2 (PTBs in green and cyan) with Numb PTB/Pon B1A2 (PTBs in blue and gray) to the A2-bound PTB. (**g**) Superimposition of representative Numb PTB in complex with Pon A2B2 (green), Pon B1A2 (orange), and Nak peptide (magenta, PDB ID: 1DDM). (**h**) The interaction details between the representative Numb PTB (grey) and B1A2 (red and light yellow) complex. Charge-charge and hydrogen-bonding interactions are highlighted by dashed lines in black. Uncropped blots are shown in Supplementary Fig. 10.

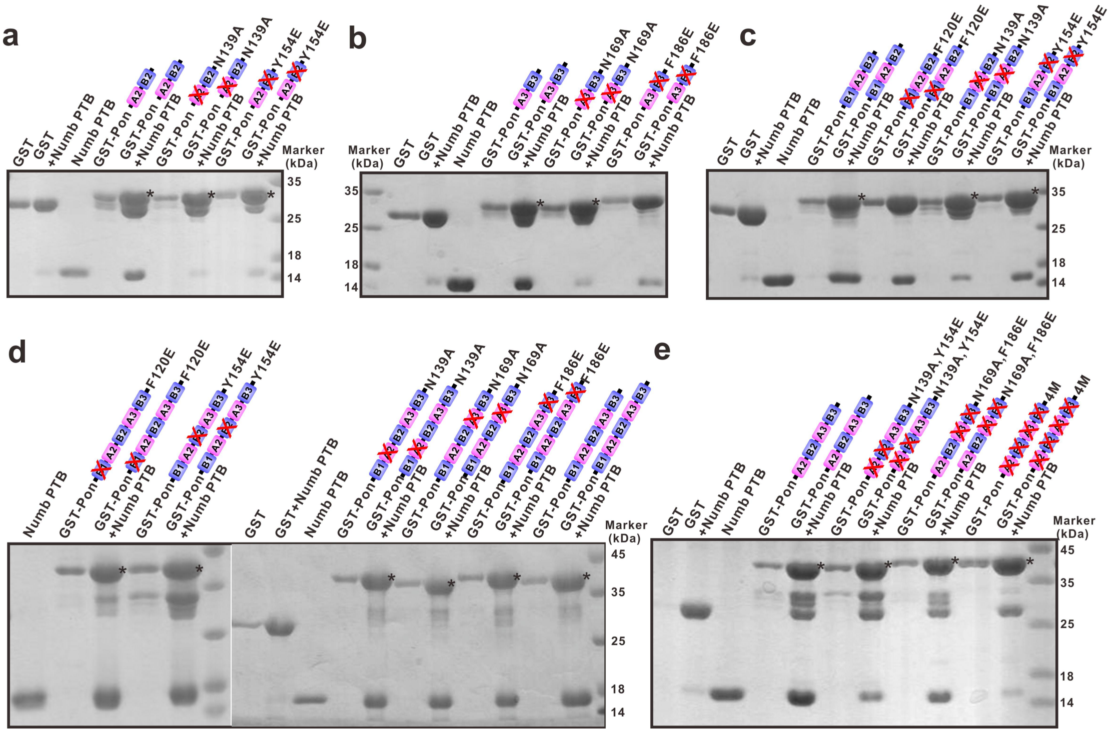

Supplementary Figure 4 Interaction between *Drosophila* Numb PTB and various Pon fragments. (**a**) Intact Pon A2B2 but not the N139A^{A2} or Y154E^{B2} mutant could specifically pull down Numb PTB. (**b**) Intact Pon A3B3 but not the N169A^{A3} or F186E^{B3} mutant could specifically pull down Numb PTB. (**c**) Pon A2 non-selectively utilizes B1 or B2 to bind to Numb PTB with high affinity. Mutation of F120E^{B1} or Y154E^{B2} caused negligible impact on the interaction between Pon B1A2B2 and Numb PTB, whereas mutation of N139A^{A2} significantly disrupted their interaction. (**d**) Multiple copies of AB repeats enhanced the binding avidity of Numb PTB to Pon. Mutation of F120E^{B1} or Y154E^{B2} caused negligible impact on the interaction between Pon B1A2B2A3B3 and Numb PTB, whereas mutation of N139A^{A2}, N169A^{A3} or F186E^{B3} significantly impaired their interaction. (**e**) Both A2B2 and A3B3 motifs are required for the efficient interaction between Numb PTB and Pon, and A2B2 contributes more than A3B3. Uncropped blots are shown in Supplementary Fig. 10.

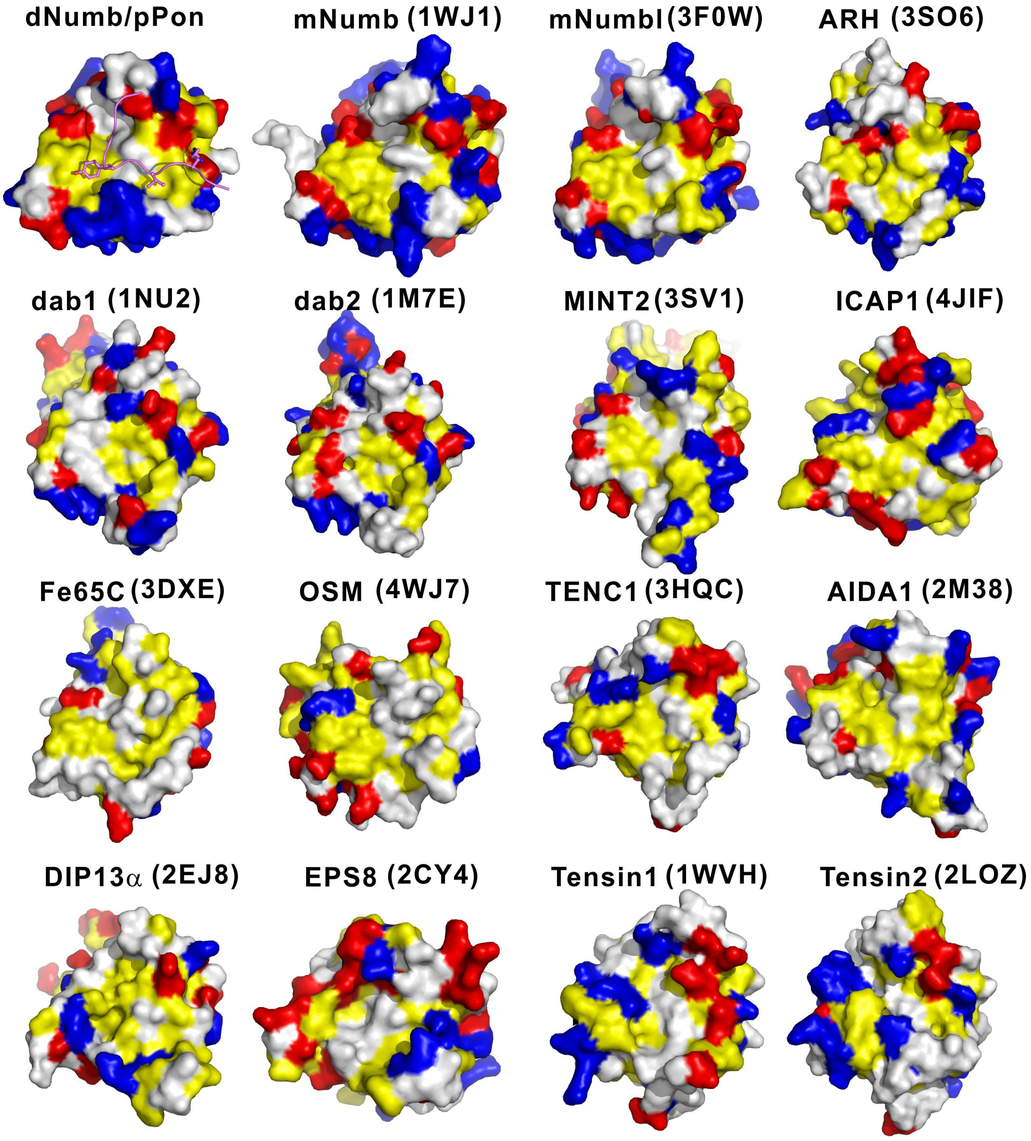

Supplementary Figure 5 The potential B motif binding pocket on PTB. Surface representation of representative PTBs from the Protein Data Bank. In the surface presentation, the hydrophobic residues are in yellow, the positively charged residues are in blue, the negatively charged residues are in red, and the rest of the amino acids are in gray. The side chains of the key residues from Pon A2B2 involved in binding to *Drosophila* Numb PTB are drawn in the ball-and-stick model.

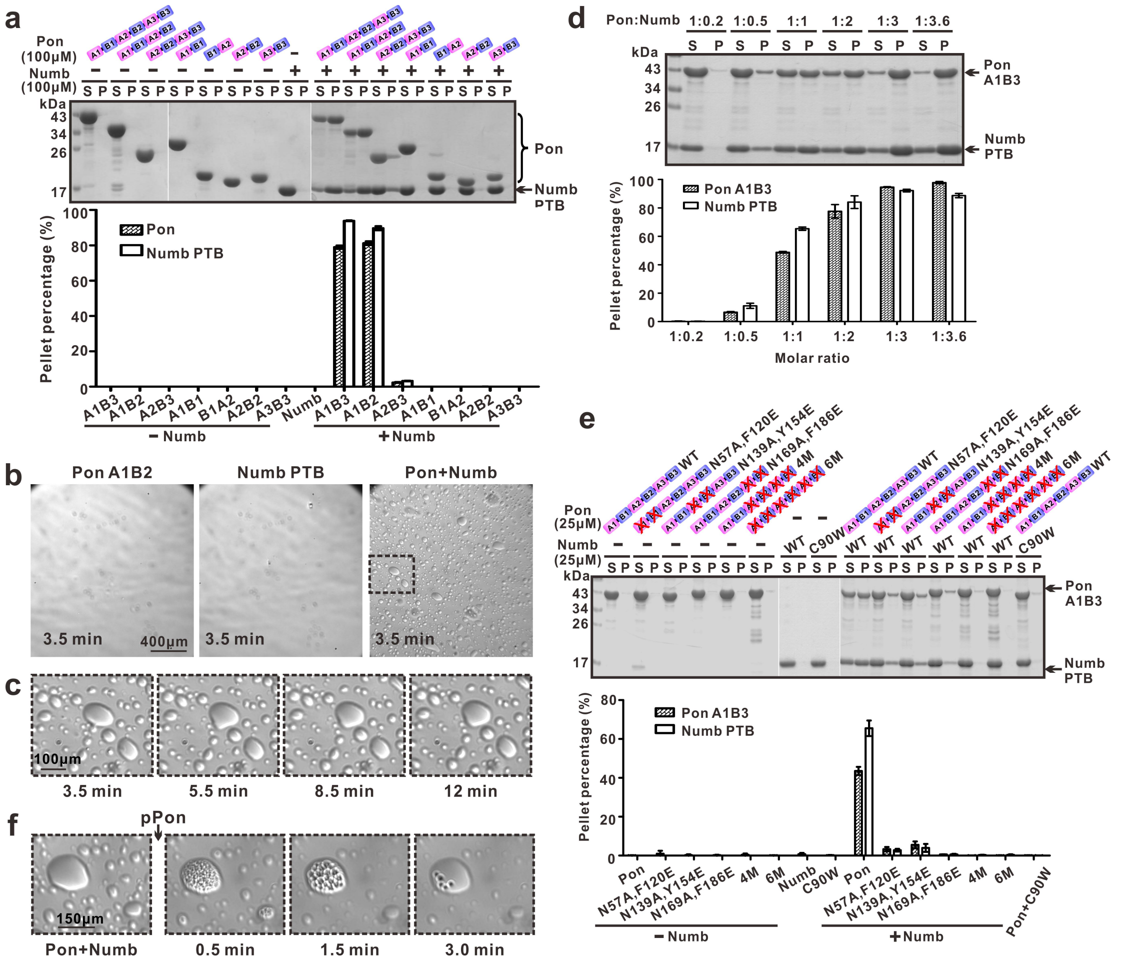

Supplementary Figure 6 Phase transition of the Numb PTB/Pon A1B3 complex *in vitro*. (**a**) Sedimentation assay of various Numb PTB/Pon fragment mixtures at 100 μM. (**b**) Isolated Numb PTB (300 μM) and Pon A1B2 (100 μM) solutions are stable and homogeneous under light microscope at RT. Mixing the two proteins with equal volumes led to formation of numerous droplets (see also Supplementary Movie 2). The images shown in the figure were acquired 3.5 min and onward after mixing. The dashed box is the region of zoomed-in analysis in **c**. (**d**) Some small droplets underwent time-dependent fusion into larger ones. (**d**) Sedimentation assay showing that the phase transition of the Numb PTB/Pon A1B3 complex is molar ratio-dependent. The concentration of Pon was fixed to 25 μM, and the molar ratios of Pon A1B3 and Numb PTB are indicated. (**e**) Numb-Pon binding deficient Pon A1B3 mutants (N57A,F120E, N139A,Y154E, N169A,F186E, 4M and 6M) or Numb PTB mutant (C90W) showing significantly impaired phase transition when compared to the WT proteins. Pon and Numb fragments were mixed at a 1:1 molar ratio and the final concentration of each protein is at 25 μM. (**f**) Time-lapse DIC images of Numb PTB (150 μM)/Pon A1B2 (50 μM) mixture showing numerous droplets at RT in a coverslip chamber. Droplets are rapidly dispersed after adding the pPon peptide. The arrow refers to the time point of adding the pPon peptide to the mixture. All statistic data in this figure represent the results from three independent batches of experiments and are expressed as mean ± SD. Uncropped gels are shown in Supplementary Fig. 10.

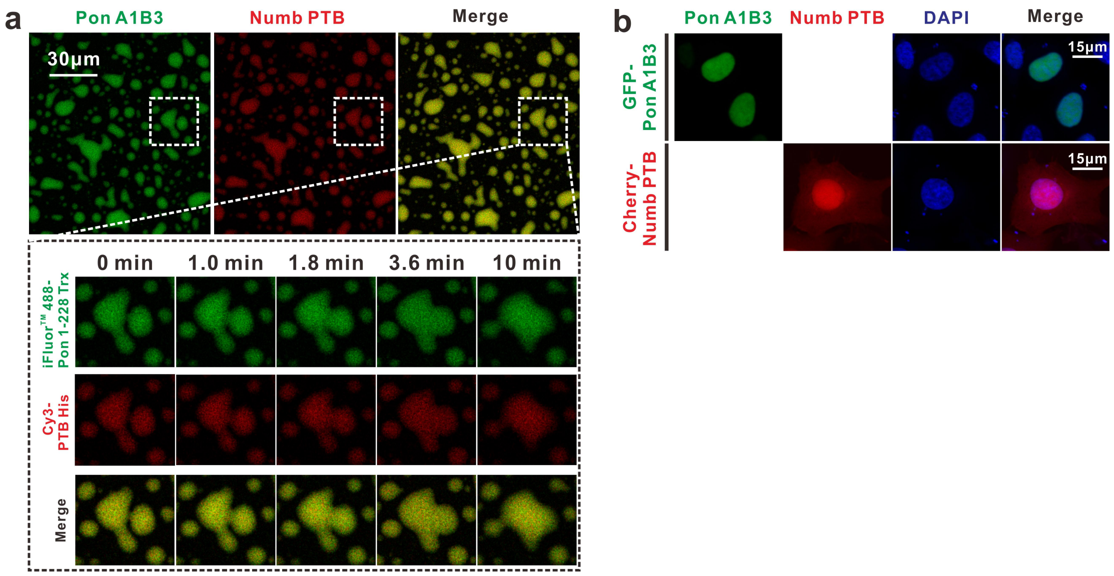

Supplementary Figure 7 Phase transition of the Numb PTB/Pon A1B3 complex in Hela cells. (**a**) The time-lapse images showing the co-localization of iFluor^{TM} 488-Pon A1B3 and Cy3-Numb PTB in the droplets with enriched concentrations. The enlarged images bellow show that small droplets can grow and merge into larger ones as time goes on (see also Supplementary Movie 4). (**b**) Representative images showing subcellular localizations of individually expressed GFP-Pon A1B3 or Cherry-Numb PTB in HeLa cells. Nuclei were stained by DAPI. All statistic data in this figure represent the results from three independent batches of experiments and are expressed as mean ± SD.

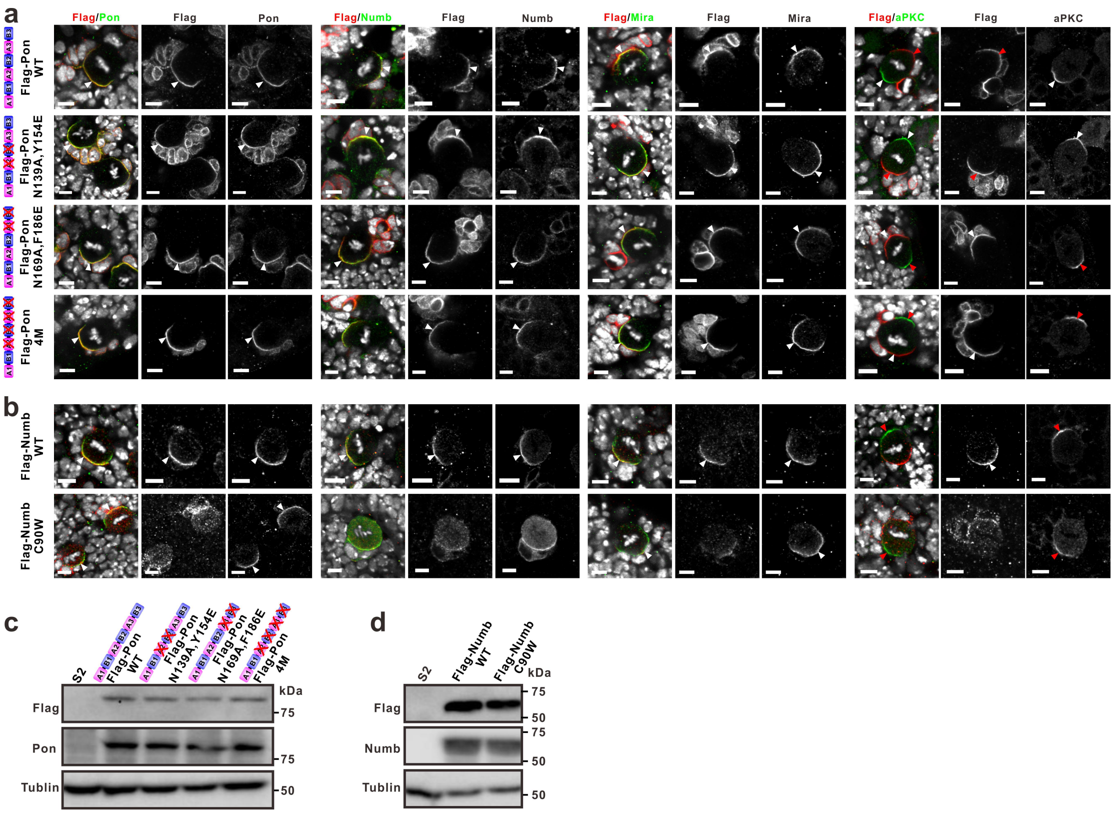

Supplementary Figure 8 Direct interaction between Pon AB motif repeats and Numb PTB is required for Numb localization during the asymmetric divisions of *Drosophila* type I larval NBs. (**a-b**) Expressing Flag-Pon WT or various mutants (**a**) or Flag-Numb WT or C90W (**b**) in wild type NBs (n=20 for each genotype) of larval brains driven by *insc-gal4*. Flag-Pon WT and mutants, and Flag-Numb WT are localized on the basal cortex. Flag-Numb C90W is largely diffused in the cytoplasm. Mira and aPKC are normally localized. ToPro-3 in white. White arrowheads point to basal cortex whereas red arrowheads indicate apical cortex. Scale bars, 5 µm. (**c-d**) Western blot showing expression of Flag-Pon (**c**) or Flag-Numb (**d**) variants in *Drosophila* S2 cells. Uncropped blots are shown in Supplementary Fig. 10.

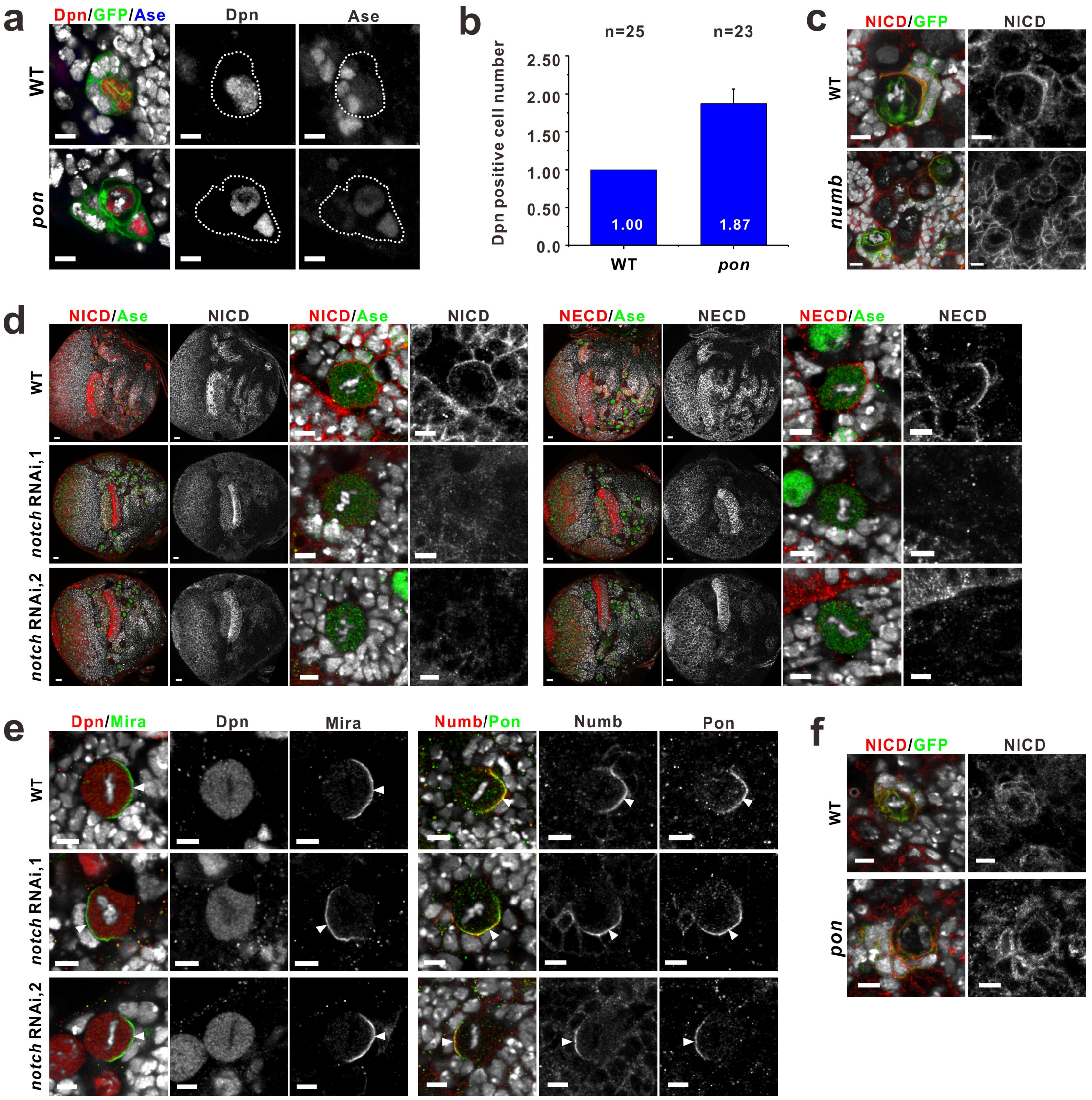

Supplementary Figure 9 Pon functions in type I NB in VNC and efficiency of Notch RNAi knockdown. (**a**) *pon* mutant NBs located in VNC region contain more Dpn-positive NB-like cells than the wild type. NBs are marked by GFP using MARCM technique. Dpn is in red, GFP in green, Ase in blue and ToPro3 in white. (**b**) Quantification of results in (**a**) showing that type I pon mutant NB clone (1.87+0.1918, n=25) in VNC contains more Dpn-positive cells compared to wt clone (1.00+0.0, n=25. $p<0.001$, mean+ SEM). Data are evaluated with Student's t-test. (**c**) The *numb* mutant NBs show normal Notch localization. (**d**) Notch expression is significantly reduced in Type I NBs (labeled by Ase expression) expressing RNAi constructs. Notch intracellular domain (NICD) or extracellular domain (NECD) is in red, Ase in green and ToPro3 in white. (**e**) Notch repression by RNAi has no impact on the localization of apical and basal proteins (red or green). (**f**) The *pon* mutant NBs show normal Notch localization. Scale bars, 25μm for the whole brain in d and 5 μm for rest NB images.

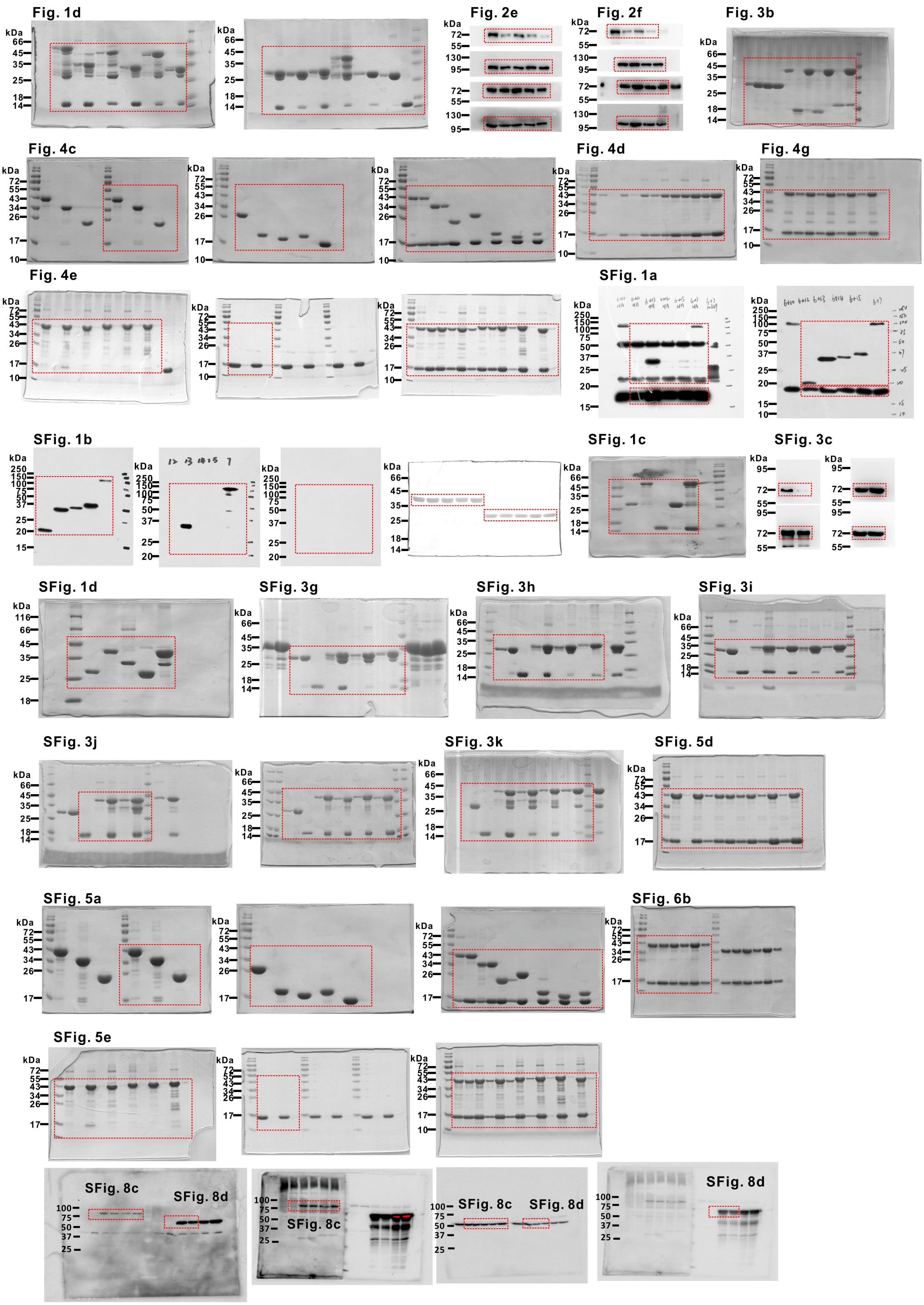

Supplementary Figure 10. Full uncropped figures of western blots and SDS-PAGE gels. Cropped regions are indicated with rectangles as appropriate.

Supplementary Table 1 Summary of the protein constructs/mutants and their performances on various assays in this study.

Constructs	Refer as to	Binding avidity	Efficiency of LLPS	Efficiency of rescuing *pon* mutant phenotype
Pon 1-670	FL WT	+ + + + +	NA	+ + + +
Pon 1-670 N57A,F120A	FL N57A,F120A	NA	NA	NA
Pon 1-670 N139A,Y154E	FL N139A,Y154E	+	NA	+
Pon 1-670 N169A,F186E	FL N169A,F186E	+ +	NA	+ +
Pon 1-670 N139A,Y154E, N169A,F186E	FL 4M	-	NA	-
Pon 1-670 N57A,F120A, N139A,Y154E,N169A,F186E	FL 6M	-	NA	NA
Pon 1-228	A1B3	+ + + + +	+ + + +	NA
Pon 1-228 N57A,F120A	A1B3 N57A,F120A	NA	+	NA
Pon 1-228 N139A,Y154E	A1B3 N139A,Y154E	NA	+	NA
Pon 1-228 N169A,F186E	A1B3 N169A,F186E	NA	+ +	NA
Pon 1-228 N139A,Y154E, N169A,F186E	A1B3 4M	NA	-	NA
Pon 1-228 N57A,F120A, N139A,Y154E,N169A,F186E	A1B3 6M	NA	-	NA
Pon 110-200	B1B3	+ + + +	NA	NA
Pon 1-162	A1B2	+ + +	+ +	NA
Pon 130-200	A2B3	+ + + +	-	NA
Pon 110-162	B1B2	+ +	NA	NA
Pon 1-130/41-130	A1B1	+	-	NA
Pon 110-148	B1A2	+ +	-	NA
Pon 130-162	A2B2	+ +	-	NA
Pon 157-200	A3B3	+ +	-	NA
Pon 145-179	B2A3	-	NA	NA
Pon 1-116	A1	-	NA	NA
Pon 124-148/130-148	A2	-	NA	NA
Pon 157-179	A3	-	NA	NA
Pon 110-130	B1	-	NA	NA
Pon 145-162	B2	-	NA	NA
Pon 177-237	B3	-	NA	NA
Numb 1-556	FL WT	+ + + + +	NA	-
Numb 1-556 C90W	FL C90W	-	NA	-
Numb 1-556 G192D	FL G192D	+	NA	NA
Numb 1-556 C90W,G192D	FL C90W,G192D	-	NA	NA
Numb 65-203	PTB	+ + + + +	+ + + +	NA
Numb 65-203 C90W	PTB C90W	-	-	NA
Numb 65-203 G192L	PTB G192L	-	NA	NA

Numb 65-203 G192D	PTB G192D	-	NA	NA
Numb 65-203 C90W,G192D	PTB C90W,G192D	-	-	NA
Nak 1431-1470	AB	+ + +	NA	NA
Nak 1431-1451	A	+	NA	NA
Nak 1451-1470	B	+	NA	NA
