# Supplementary material for: Basal condensation of Numb and Pon complex via phase transition during Drosophila neuroblast asymmetric division
Source: Nat Commun. 2018 Feb 21;9:737. doi: 10.1038/s41467-018-03077-3 (PMC5821850; doi:10.1038/s41467-018-03077-3)
Supplement: Supplementary file 3 — Description of Additional Supplementary Files [file 41467_2018_3077_MOESM3_ESM.pdf]

Description of Additional Supplementary Files

File Name: Supplementary Movie 1
Description: Mixing Numb PTB (300 µM) and Pon A1B3 (100 µM) with equal volumes led to formation of numerous droplets. The movie was acquired 2 min and onward after mixing.

File Name: Supplementary Movie 2
Description: Mixing Numb PTB (300 µM) and Pon A1B2 (100 µM) with equal volumes led to formation of numerous droplets. The movie was acquired 2 min and onward after mixing.

File Name: Supplementary Movie 3
Description: The pPon peptide binding-induced dispersion of the Numb PTB/Pon A1B3 droplets.

File Name: Supplementary Movie 4
Description: Time-dependent fusion of small Cy3-Numb PTB/iFluorTM 488-Pon A1B3 droplets into larger ones *in vitro*.

File Name: Supplementary Movie 5
Description: Fluorescence signal recovery of Cy3-Numb PTB *in vitro*.

File Name: Supplementary Movie 6
Description: Fluorescence signal recovery of GFP-Pon A1B3 in a HeLa cell co-expressing with Cherry-Numb PTB.
